# A framework for production of systematic review based briefings to support evidence-informed decision-making

**DOI:** 10.1186/2046-4053-1-32

**Published:** 2012-07-09

**Authors:** Duncan Chambers, Paul Wilson

**Affiliations:** 1 Centre for Reviews and Dissemination, University of York, Heslington, York, YO10 5DD, UK

**Keywords:** Evidence briefings, Decision-making, Knowledge translation

## Abstract

**Background:**

We have developed a framework for translating existing sources of synthesized and quality-assessed evidence, primarily systematic reviews, into actionable messages in the form of short accessible briefings. The service aims to address real-life problems in response to requests from decision-makers.

Development of the framework was based on a scoping review of existing resources and our initial experience with two briefing topics, including models of service provision for young people with eating disorders. We also drew on previous experience in dissemination research and practice. Where appropriate, we made use of the SUPporting POlicy relevant Reviews and Trials (SUPPORT) tools for evidence-informed policymaking.

**Findings:**

To produce a product that it is fit for this purpose it has been necessary to go beyond a traditional summary of the available evidence relating to effectiveness. Briefings have, therefore, included consideration of cost effectiveness, local applicability, implications relating to local service delivery, budgets, implementation and equity. Our first evidence briefings produced under this framework cover diagnostic endoscopy by specialist nurses and integrated care pathways in mental healthcare settings.

**Conclusions:**

The framework will enable researchers to present and contextualize evidence from systematic reviews and other sources of synthesized and quality-assessed evidence. The approach is designed to address the wide range of questions of interest to decision-makers, especially those commissioning services or managing service delivery and organization in primary or secondary care. Evaluation of the use and usefulness of the evidence briefings we produce is an integral part of the framework and will help to fill a gap in the literature.

## Background

Producers of systematic reviews use various methods to make their findings more accessible to decision-makers, including plain language summaries, structured critical abstracts, overviews of reviews on a particular topic, and briefings that combine systematic reviews with other evidence sources [1,2]. The process of adapting research evidence to meet the needs of decision-makers and encourage them to use evidence is sometimes referred to as ‘knowledge translation’. As part of a larger research project, we are providing a knowledge translation service to National Health Service (NHS) decision-makers, translating existing sources of synthesized and quality-assessed evidence, primarily systematic reviews, into actionable messages [3]. The evidence is presented in the form of short (typically around 3,000 words), accessible evidence briefings with a bullet point summary of key findings and implications. The service was initially aimed at commissioners of healthcare services but has also been utilized to support decisions relating to service delivery and organization in acute and community mental health care settings.

In order to produce a product that it is fit for this purpose, it has been necessary to go beyond a traditional summary of the available evidence relating to effectiveness [4,5]. Briefings have, therefore, included consideration of cost effectiveness, local applicability, implications relating to local service delivery, budgets, implementation and equity. Initial evidence briefings covered cognitive behavior therapy (CBT) for schizophrenia and alternatives to in-patient admission for young people with eating disorders. These briefings were produced using an intuitive *ad hoc* process. While they were generally well received, it became apparent that we needed to develop a standardized process to clarify and confirm the question(s) to be considered and the methods used to address them. In this paper we present the framework that we have developed and are currently testing.

## Methods

Development of the framework was based on a scoping review of existing resources [1] and our initial experience of a range of briefing topics, including CBT for schizophrenia and models of service provision for young people with eating disorders. We also drew on previous experience in this area with the renowned *Effective Health Care* bulletin series [6], and experience in dissemination research and practice. Where appropriate, we made use of the SUPPORT tools for evidence-informed policymaking [7]. The methods we use for producing evidence briefings are described below

## Results

### Generating topics

Briefings are produced in response to requests from NHS decision-makers who require an independent assessment of evidence to inform a decision. The rationale for this was based on our experience that a briefing produced in response to a real-life problem (service reconfiguration for eating disorders) had an impact that other pilot briefings lacked. The service was initially aimed at commissioners of healthcare services but in principle the framework could also cover a wide range of other decisions in the areas of clinical effectiveness and service delivery and organization.

### Clarifying the research question

On receiving a request, the first step is to arrange a meeting to clarify the question(s) to be addressed. Wherever possible this will be a face to face meeting with those involved in the decision from different perspectives (for example, managers, clinicians and potentially patient/carer representatives). This is important because direct contact between decision-makers and researchers has been identified as a facilitator of use of research evidence [4,8].

The objectives of the meeting are to:

· clarify the issues to be addressed in terms of population, intervention, comparator and outcomes (PICO). If the research question as originally framed appears to be excessively broad, it may be necessary to modify the scope or break the question down into a number of more specific questions;

· discuss the background to the decision and obtain as much relevant contextual information as possible;

· agree a timescale for production and review of the evidence briefing.

**Figure 1:**
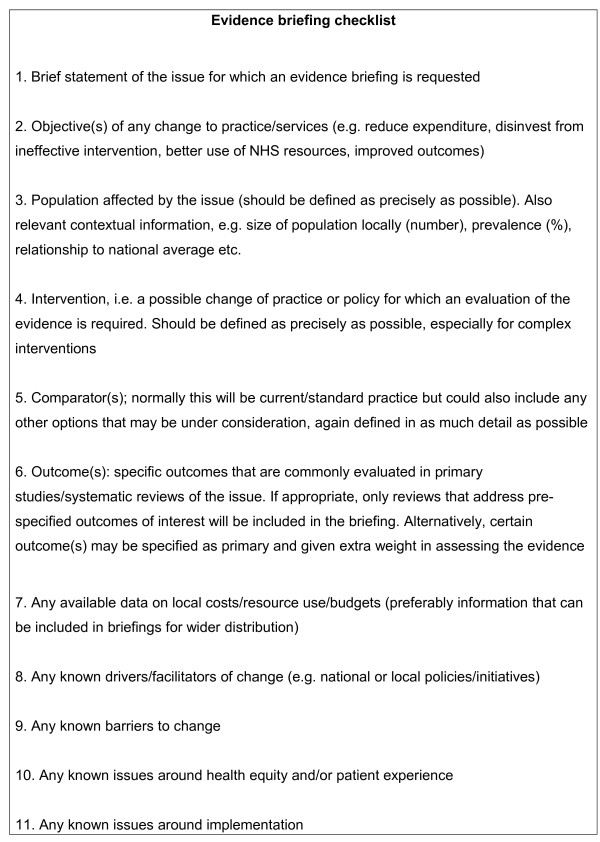
Checklist to clarify the research question

Our checklist of information to clarify the research question and establish the local context is presented in Figure 1. We will keep the content of the checklist under review and amend it if necessary in the light of further experience with working with local decision-makers.

At this meeting we also make clear our intent to make the final evidence briefing publicly available and aim to agree with the ‘customer’ on an appropriate timeframe for making a final version available to the wider NHS community. Evidence briefings are treated as confidential during the process of production and review. The research question will normally involve a comparison of the evidence base for two or more interventions (including models of delivery and/or organization of care). Our working assumption is that the decision-makers have framed the problem and identified appropriate options before approaching us and it is not our primary role to address any other alternatives that may exist. However, the possibility that the question may be modified as a result of our research should not be ruled out. If at any point it appears that the question cannot be appropriately answered using existing evidence sources (that is, a new systematic review or primary research is required) we will inform the ‘customer’ of this and stop work on the evidence briefing.

### Systematic reviews

Searches for relevant evidence are performed by the researcher responsible for the briefing, with the involvement of an information specialist for more complex topics. The primary sources of evidence about effectiveness are systematic reviews. Reviews are identified by searching the following sources:

· Database of Abstracts of Reviews of Effects (DARE) for quality-assessed systematic reviews of interventions;

· Cochrane Database of Systematic Reviews;

· NHS Health Technology Assessment (HTA) programme reports;

· Centre for Reviews and Dissemination (CRD) HTA database;

· National Institute for Health and Clinical Excellence (NICE) guidelines (for systematic reviews performed to support guideline recommendations).

DARE focuses on systematic reviews that evaluate the effects of health and social care interventions and the delivery and organization of care. The DARE process involves extensively searching for, identifying, and critically appraising the global stock of systematic reviews. DARE currently provides access to over 25,000 systematic reviews and this content is also supplied to The Cochrane Library, PubMed Health, NHS Evidence and Health Systems Evidence. Other sources of systematic reviews and HTA reports (for example, Rx for Change [9], and the McMaster Health Forum’s Health Systems Evidence [10]) may be searched if appropriate. We do not normally search for primary research studies but will do so if necessary (for example, to update existing systematic reviews or if there are important gaps in the evidence available from systematic reviews).

### Economic evidence

Economic evaluations are identified from the following sources:

· NHS Economic Evaluation Database (NHS EED);

· NICE guidelines (for economic modeling studies performed to support guideline recommendations and other economic evidence):

· NHS HTA program reports and CRD HTA database (for health technology assessments incorporating economic evaluation).

### Assessment of quality

For systematic reviews and economic evaluations derived from DARE and NHS EED, we are able to make use of an existing critical appraisal (structured abstract) in most cases. If a critical abstract has not been written, we critically appraise the study using standard DARE and NHS EED methods. Cochrane Reviews and NHS HTA program reports are considered high-quality evidence sources and are not formally appraised as part of the DARE production process. We will, however, assess the quality and reliability of such reviews where they are included in an evidence briefing.

If systematic review evidence is limited and the best evidence clearly comes from one or two primary studies, we will critically appraise this evidence using the approach of the Cochrane Effective Practice and Organization of Care (EPOC) Group [11].

### Local context

Our briefings relate the evidence to the local setting. As a minimum, we assess generalizability of the evidence to the UK NHS (to what extent were studies in the included systematic reviews and economic evaluations conducted in similar populations/settings?) at the local as well as the national level; potential impact on outcomes locally (based on effect measures reported in or calculated from systematic reviews); and state any implications for local service delivery and budgets. We use items 7 to 10 of our checklist to work with the customer to identify any evidence related to the local context. These are supplemented by interrogation of any national/local policy and guidance documents, prevalence data or episode statistics that are relevant to the question under consideration. Any variations in the availability, quality or results of local evidence are assessed and described using an approach based on previous experience of producing national guidance on commissioning cancer services but one that is also similar to the approach of the SUPPORT Collaboration [12].

### Health equity

It is important to assess any implications of changes to practice or service delivery for health equity. Our preferred approach follows that developed by the SUPPORT Collaboration [13] and involves consideration of the following questions:

· Which groups or settings are likely to be disadvantaged in relation to the option being considered?

· Are there plausible reasons for anticipating differences in the relative effectiveness of the option for disadvantaged groups or settings?

· Are there likely to be different baseline conditions across groups or settings such that the absolute effectiveness of the option would be different, and the problem more or less important, for disadvantaged groups or settings?

· Are there important considerations that should be made when implementing the option to ensure that inequities are reduced, if possible, and that they are not increased?

However, we recognize that this information may not always be available from systematic reviews and is likely to need to be supplemented or replaced by information gathered locally, using documents produced by or relevant to the NHS, such as Joint Strategic Needs Assessments and equity audits.

### Implementation

We also attempt to assess the likely ease of implementation of any changes to practice or service delivery. This involves consideration of issues such as the time and resources required to implement change, the numbers of services and staff affected, and the likely attitudes of relevant stakeholders. To date we have used this informal approach rather than the more comprehensive approach, including consideration of implementation strategies, developed by the SUPPORT Collaboration [14]. Initial experience suggests that this simpler approach is sufficient to meet the needs of NHS decision-makers, but we will keep this issue under review.

In addition, as health care resources are finite there may also be a need to consider the costs and benefits of investment in implementation itself. Implementation efforts compete with other health-care programs for limited health-care resources, so it is, therefore, important that we determine whether implementation is actually worthwhile. We have been exploring the feasibility of applying a framework for assessing the cost-effectiveness of quality improvement efforts [15], but recognize that this is an area in need of methodological development.

### Briefing format

**Figure 2:**
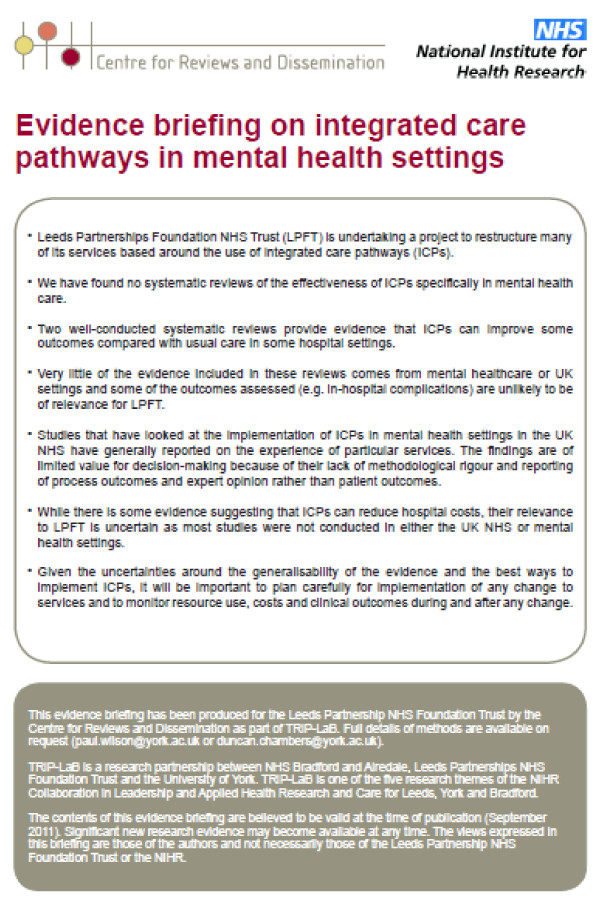
Example of an evidence briefing.

The precise format depends on the topic but will normally include:

· Front page bullet point summary of main messages (Figure 2).

· Background section describing local context and topic to be addressed.

· Methods section.

· Evidence base for effectiveness. Summary of existing systematic reviews and their findings; critical appraisal of the strength of the evidence and methodological rigor of the review(s); assessment of generalizability. Critical appraisal based as far as possible on information contained in the reviews themselves and existing appraisals, such as DARE abstracts.

· Evidence base for cost-effectiveness. Summary of existing economic evaluations/models and their findings; critical appraisal of strength of the evidence and methodological rigor of the studies. Critical appraisal based as far as possible on information contained in the studies themselves and existing appraisals such as NHS EED abstracts.

· Potential implications. Based on the evidence presented, what are the possible implications of any decision to change practice or service delivery in the local NHS setting in terms of quality of care; patient and process outcomes; cost savings or better use of resources; and health equity? Implementation issues will also be considered.

· References.

Additional information, such as search strategies and data extraction tables/evidence profiles, is made available as appendices or on request from the authors.

### Peer review/quality control

As a minimum, briefings are reviewed and edited by a second researcher and representative(s) of the customer organization. To date, peer review has been undertaken by researchers independent of the project team.

### Evaluation

**Figure 3:**
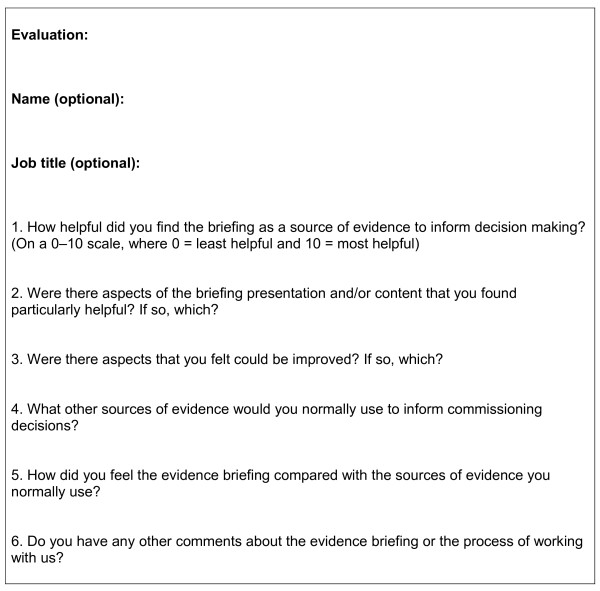
Evidence briefing evaluation questionnaire

Evaluation of use, usefulness and impact is an important part of the process of evidence briefing production and interaction with decision-makers. The briefing on models of service provision for adolescents with eating disorders (produced before we developed this framework) was evaluated using a brief questionnaire (Figure 3) that went to everybody who received the briefing. While the feedback was positive, the sample size was very small [16].

Our first evidence briefings produced under this framework cover diagnostic endoscopy by specialist nurses, integrated care pathways in mental healthcare settings and drug treatments for patients with functional class II pulmonary hypertension. For full details of all evidence briefings, see the project web page [17]. At the time of writing, these briefings are part of ongoing decision making processes. A feedback process will be instigated once the deliberations have concluded. We will continue to evaluate the service on an ongoing basis.

## Discussion

The framework presented in this paper is intended to enable researchers to present and contextualize evidence from systematic reviews and other sources of synthesized and quality-assessed evidence. The approach is designed to address the wide range of questions of interest to decision-makers, especially those commissioning services or managing service delivery and organization in primary or secondary care. As such, the framework attempts to go beyond the types of questions normally addressed in systematic reviews of effectiveness. We aim to build relations with decision-makers through initial face to face meetings, followed by continued contact (face-to-face or email) to clarify the issue or question to be addressed. The use of the checklist (Figure 1) enables us to ensure that a common understanding of the question to be addressed is achieved. The checklist takes a broad approach to defining the question, especially for aspects other than effectiveness and cost-effectiveness. For example, there could be overlap between the concepts covered by questions 8, 9 and 11, but having three separate questions allows for differences in understanding of terms like ‘barriers’, ‘facilitators’ and ‘implementation’.

While the approach appears promising, our experience with producing briefings using this framework has been limited to date. Collection of feedback from decision-makers is an important part of the process and will help us to refine our approach further over time. We are aware of the need to develop the peer review system and possibly to involve a wider range of experts, particularly for assessing aspects other than clinical and cost-effectiveness.

As a matter of policy, we are currently limiting production of evidence briefings to questions brought to us by decision-makers, rather than proactively seeking to identify topical and important issues. This could be seen as either a strength or a weakness in our approach. Analyzing real problems in collaboration with those directly affected should mean that research evidence is more likely to be used and have an impact on decision-making. Systematic reviews suggest that the interaction of decision-makers and researchers promotes uptake of research evidence [8]. On the other hand, engagement with decision-makers has historically been a challenge for this type of service [1]. The challenge for us has been to generate enough topics initially to get the service off the ground, particularly at a time of change and uncertainty in the English NHS. Demand for the service is expected to increase once the transition to a system of clinically-led commissioning has occurred because there will be more commissioning bodies with varying levels of expertise and access to resources to support evidence-informed decision-making.

Services that synthesize systematic reviews with other research evidence and context-specific information to answer a specific question are defined as ‘policy briefs’ in the taxonomy developed by Lavis [2]. These services differ from ours in being primarily aimed at government or regional level decision-making. Another point of difference is that in the model of policy briefs described by Lavis and the SUPPORT Collaboration, it appears that the people producing the brief identify possible options to solve the problem being addressed, even if they do not make recommendations [18]. Our preferred approach is to evaluate solutions already under consideration by decision-makers.

Some new services of this type have started since we did the searches for our scoping review, demonstrating a wide current interest in optimizing the use of systematic reviews by decision-makers. This may be related to the increasing pressure to make the best use of limited resources for healthcare in both developed and developing countries. The closest parallel to our service has been developed by the Ottawa Hospital Research Institute, Canada, working with the Champlain Local Health Integration Network, a commissioner of healthcare services [19]. This service, known as ‘Knowledge to Action’, has produced 16 systematic review-based evidence summaries at the time of writing. However, these summaries appear to be mainly overviews of the systematic review literature (including quality assessment using AMSTAR) with less emphasis on the consideration of context, the potential cost impact, implementation and health equity that is integral to our framework. In Africa, rapid response evidence services for national-level decision-makers, based on systematic reviews where possible, have been set up in Uganda and Burkina Faso as part of the EVIPNet (Evidence-Informed Policy Network) program supported by the World Health Organization [20].

As noted previously [1], there have been few formal published evaluations of these services, although it is likely that service providers have gathered substantial amounts of information that is not in the public domain. This reinforces the need for us to evaluate the perceived usefulness and use of the briefings that we produce. The eating disorders briefing was evaluated by means of a brief questionnaire. More sophisticated approaches to evaluation could be developed although it may be unrealistic to expect high response rates from NHS commissioners and clinicians.

Further developments could include incorporation of local data supplied to us by the organization requesting the briefing and extraction of data from the wide range of resources available through, for example, the NHS Information Centre. If we were to attempt the latter, systematic and transparent methods for searching and using the data would need to be developed.

Peer review is another area that we are seeking to develop further and is especially important in relation to adapting the briefings for wider audiences. As we decide how best to balance the need for rapid information to support decision-making against the time required for rigorous peer review, it may be that (as with *Effective Health Care*), we will be able to recruit a core group of responsive peer reviewers. Post-publication peer review is also possible, with readers invited to submit comments for response.

Given that we are going beyond the boundaries of standard systematic reviews/HTAs or even other evidence briefing services, it will be particularly important to determine the feasibility of assessing potential impacts on equity and implementation using the approach outlined in this framework, or whether further expert input will be required.

## Conclusions

We have developed a framework designed to use systematic reviews, economic evidence and relevant contextual data to support evidence-informed decision-making, particularly in relation to commissioning of healthcare services and models of service delivery and organization. The framework is based on knowledge of existing services and builds on our previous experience with working with decision-makers in the English NHS. Evaluation of the use and usefulness of the evidence briefings we produce is an integral part of the framework and will help to fill a gap in the current literature.

## Abbreviations

CBT, Cognitive behavior therapy; CRD, Centre for Reviews and Dissemination; DARE, Database of Abstracts of Reviews of Effects; EPOC, Effective Practice and Organization of Care; EVIPNet, Evidence-Informed Policy Network; HTA, Health Technology Assessment; NHS, National Health Service; NHS EED, NHS Economic Evaluation Database; NICE, National Institute for Health and Clinical Excellence; PICO, Population, Intervention, Comparator and Outcomes.

## Competing interests

The authors have no competing interests to declare.

## Authors’ contributions

DC and PW contributed equally to the work reported in this paper and to the writing of the paper. Both authors read and approved the final manuscript.

## Authors’ information

DC and PW are Research Fellows at the Centre for Reviews and Dissemination (CRD), University of York, UK. PW manages the CRD knowledge translation service and is a co-applicant of the TRiP-LaB (Translating Research into Practice in Leeds and Bradford) research theme which supported much of the work described in this paper. DC is a TRiP-LaB Research Fellow based at CRD.
